# The common rs9939609 variant of the fat mass and obesity-associated gene is associated with obesity risk in children and adolescents of Beijing, China

**DOI:** 10.1186/1471-2350-11-107

**Published:** 2010-07-05

**Authors:** Bo Xi, Yue Shen, Meixian Zhang, Xin Liu, Xiaoyuan Zhao, Lijun Wu, Hong Cheng, Dongqing Hou, Klaus Lindpaintner, Lisheng Liu, Jie Mi, Xingyu Wang

**Affiliations:** 1 Department of Epidemiology, Capital Institute of Pediatrics, 2 Ya Bao Road, 100020 Beijing China; 2 Laboratory of Human Genetics, Beijing Hypertension League Institute, 100043 Beijing China; 3 Roche Center for Medical Genomics, F. Hoffmann-La Roche, Basel, Switzerland

## Abstract

**Background:**

Previous genome-wide association studies for type 2 diabetes susceptibility genes have confirmed that a common variant, rs9939609, in the fat mass and obesity associated (*FTO*) gene region is associated with body mass index (BMI) in European children and adults. A significant association of the same risk allele has been described in Asian adult populations, but the results are conflicting. In addition, no replication studies have been conducted in children and adolescents of Asian ancestry.

**Methods:**

A population-based survey was carried out among 3503 children and adolescents (6-18 years of age) in Beijing, China, including 1229 obese and 2274 non-obese subjects. We investigated the association of rs9939609 with BMI and the risk of obesity. In addition, we tested the association of rs9939609 with weight, height, waist circumference, waist-to-height ratio, fat mass percentage, birth weight, blood pressure and related metabolic traits.

**Results:**

We found significant associations of rs9939609 variant with weight, BMI, BMI standard deviation score (BMI-SDS), waist circumference, waist-to-height ratio, and fat mass percentage in children and adolescents (*p *for trend = 3.29 × 10^-5^, 1.39 × 10^-6^, 3.76 × 10^-6^, 2.26 × 10^-5^, 1.94 × 10^-5^, and 9.75 × 10^-5^, respectively). No significant associations were detected with height, birth weight, systolic and diastolic blood pressure and related metabolic traits such as total cholesterol, triglycerides, HDL-cholesterol, LDL-cholesterol and fasting plasma glucose (all *p *> 0.05). Each additional copy of the rs9939609 A allele was associated with a BMI increase of 0.79 [95% Confidence interval (CI) 0.47 to 1.10] kg/m^2^, equivalent to 0.25 (95%CI 0.14 to 0.35) BMI-SDS units. This rs9939609 variant is significantly associated with the risk of obesity under an additive model [Odds ratio (OR) = 1.29, 95% CI 1.11 to 1.50] after adjusting for age and gender. Moreover, an interaction between the *FTO* rs9939609 genotype and physical activity (*p *< 0.001) was detected on BMI levels, the effect of rs9939609-A allele on BMI being (0.95 ± 0.10), (0.77 ± 0.08) and (0.67 ± 0.05) kg/m^2^, for subjects who performed low, moderate and severe intensity physical activity.

**Conclusion:**

The *FTO *rs9939609 variant is strongly associated with BMI and the risk of obesity in a population of children and adolescents in Beijing, China.

## Background

The increasing prevalence of obesity among adults and children as well as related conditions including type 2 diabetes mellitus [1], cardiovascular diseases [2], hypertension [2], and stroke [3] have become major health concerns. In China, the nationwide prevalence of overweight and obesity among adults has been estimated to be 22.8% and 7.1%, respectively [4]. A dramatic increase in the number of overweight and obese children ages 7-18 years has been observed; a recent study reported that in northern coastal cities, the combined prevalence had reached 32.5% for boys and 17.6% for girls in 2005 [5]. Large population-based studies have been conducted to identify common genetic variants that contribute to obesity. Several promising genes have been reported to be associated with a variation in body mass index (BMI) in a monogenic or polygenic manner: proprotein convertase subtilisin/kexin type 1 (*PCSK1*) [6], melanocortin 4 receptor (*MC4R*) [7], catenin beta-like 1 (*CTNNBL1*) [8], ectonucleotide pyrophosphatase 1 (*ENPP1*) [9], and beta 2-adrenergic receptor (*ADRB2*) [10]. A previously reported population-based meta-study involving as many as 38759 individuals implemented a genome-wide search for type 2 diabetes susceptibility genes. Frayling *et al. *[11] identified a common variant (rs9939609) in the fat mass and obesity associated (*FTO*) gene region that represents a T to A change in the first intron of the *FTO *gene on chromosome 16. They found that there were significant associations of this single nucleotide polymorphism (SNP) with BMI (*p *= 3 × 10^-35^) and the risk of obesity [Odds ratio (OR) = 1.67, 95% Confidence interval (CI) 1.47 to 1.89, *p *= 1 × 10^-14 ^for the homozygous condition] in a European adult population. The A allele was also associated with an increased BMI in European children from age 7 and older (e.g., at age 11 years, *p *= 7 × 10^-9^), and conferred an increased risk of childhood obesity (e.g., OR per A allele at age 11 years = 1.35, 95% CI 1.14 to 1.61, *p *= 6 × 10^-4^) [11]. However, replication studies in Asian adult populations yielded inconsistent results. Li *et al. *[12] reported lack of association of *FTO *rs9939609 with risk of obesity in a Chinese population (*n *= 3210, *p *= 0.96). Subsequently, Chang *et al. *[13] reported a positive association of rs9939609 with the risk of obesity and BMI in a population of Taiwan, China (*n *= 2248, *p *= 7 × 10^-4^). Hotta *et al. *[14] reported that the *FTO *rs9939609 variant is associated with severe obesity in the Japanese (*n *= 2454, *p *= 2 × 10^-5^), and a study from Singapore (*n *= 2919) also reported significant associations of rs9939609 with BMI and obesity (*p *< 0.0001) [15].

To date, there are no replication studies reported in children and adolescents of Asian ancestry. We genotyped rs9939609 in children and adolescents that participated in the epidemiological Beijing Child and Adolescent Metabolic Syndrome (BCAMS) study to assess the association of this SNP with BMI and related traits. We tested the association of rs9939609 with BMI as a continuous trait and with the risk of obesity as a dichotomised trait. Other obesity-related anthropometric and metabolic parameters were also tested for their association with rs9939609.

## Methods

### Subjects

Subjects were recruited from the cross-sectional population-based Beijing Child and Adolescent Metabolic Syndrome (BCAMS) study [16]. The study included completion of a questionnaire, a medical examination, anthropometric measurements, and finger capillary blood tests from a representative sample of Beijing school-age children (*n *= 19593, ages 6-18 years, 50% boys) between April and October 2004. Anthropometric measurements included weight, height, waist circumference (WC), and fat mass percentage (FMP). BMI (equal to weight/height^2^) and FMP were used as measures of general adiposity, whereas WC and waist-to-height ratio (WHtR, equal to WC/height) were used as measures of central adiposity.

The questionnaires were completed by parents or guardians if the participant was 12 years or younger and by the subject if the participant was 13 years or older. Information regarding birth weight and physical activity were collected. The estimation of physical activity was recorded as the total days per week with at least 30 mins per day participating in extracurricular physical activities such as cycling, roller-skating, running, swimming, dancing, team sports and tennis, etc.. The intensity of physical activity was divided into 3 categories as low (< 3 days/week), moderate (3-5 days/week) and severe (≥ 5 days/week).

Within this large group of school-age children, 4500 were screened out as the presence of any one of the following traits: overweight based on BMI standards, increased cholesterol (≥ 5.2 mM), increased triglycerides (≥ 1.7 mM) or increased glucose (≥ 5.6 mM). A parallel reference population of 1045 school-age children was also studied. Within these two groups, 2544 (BCAMS) and 981 (reference) children and adolescents were recruited for blood samples [17]. Of the 3525 total subjects, BMI values or genotyping information were not available for 22 subjects, and these individuals' records were deleted from the database. In our study, 1229 obese children (786 boys, 64.0%) and 2274 non-obese (including normal and overweight) children (995 boys, 43.8%) were diagnosed by the Chinese age- and gender-specific BMI standards [18]. The BCAMS study was approved by the ethics committee and institutional review board of the Capital Institute of Pediatrics (CIP). All participating children and adolescents as well as their parents gave written informed consent under protocols provided by the CIP that clearly stated that blood samples would be used for scientific research purposes, including genetic studies. A health questionnaire was administered to each participant at the same time.

### Measurement of anthropometric parameters

All instruments were validated following the manufacturers' protocols [19]. Height without shoes was measured using wall-mounted stadiometers to the nearest 0.1 cm. Body weight was measured with underwear and no shoes to the nearest 0.1 kg using beam scales with a maximum weight of 140 kg. BMI (kg/m^2^) was calculated as body weight/height^2^. BMI standard deviation score (BMI-SDS) for each participant was calculated based on Chinese National Survey on Students Constitution and Health in 2000[18]. WC was measured midway between the lowest rib and the superior border of the iliac crest with an inelastic measuring tape at the end of normal expiration to the nearest 0.1 cm. FMP was assessed by bioelectrical impedance analysis (TANITA TBF-300A). Blood pressure (BP) was measured with mercury manometer. Systolic BP (SBP) was indicated by Korotkoff's first phase and diastolic BP (DBP) by Korotkoff's fourth phase.

Fasting plasma glucose (FPG) was determined by the glucose oxidase method. Serum total cholesterol (TC) and triglyceride (TG) concentrations were determined using standard enzymatic methods. HDL-cholesterol (HDL-C) and LDL-cholesterol (LDL-C) were measured directly. The serum lipid levels and FPG were assayed using the Hitachi 7060C Automatic Biochemistry Analysis System.

### Genotyping

The rs9939609 SNP of the *FTO *gene was genotyped by allele-specific real-time polymerase chain reaction (RT-PCR) [20] using an ABI 5700 Real Time PCR Instrument (Applied Biosystems, Foster City, California, United States). Genomic DNA was isolated from peripheral white blood cells using the salt fractionation method. DNA samples were genotyped for a single SNP using an equal aliquot of sample in two allele-specific PCR reactions, each containing one allele-specific primer (AGACTATCCAAGTGCATCACA or AGACTATCCAAGTGCATCACT) and a common primer (ATTCTAGGTTCCTTGCGACT). PCR reactions with an allele-specific primer that is mismatched to the template alleles will be prevented or delayed when monitored in real-time with SYBR Green I^®^. For each amplification, a fluorescence threshold near the baseline fluorescence was used to calculate a cycle threshold (Ct) value. This value was then used to determine the genotype of the sample. PCR was carried out on a GeneAmp 5700 Sequence Detector with an enzyme heat-activation step of 12 min at 95°C, followed by 45 cycles of two-step amplification (30 seconds at 95°C, 30 seconds at 58°C and an on-board dissociation run from 60°C to 95°C). The genotype was determined from the Ct values obtained with the GeneAmp 5700 SDS software. The genotyping call rate was 95.73% for the cohort after the first genotyping reaction, and we obtained 99.97% of the samples' genotypes by re-genotyping. We estimated the genotyping error rate at < 1% by validating 100 random samples of known genotype in additional reactions.

### Statistical analysis

Quantitative variables were expressed as means ± standard deviation (SD), or means ± standard error (SE), and differences between groups were assessed with the Student's *t*-test. Categorical variables were represented as percentages and were tested by the χ^2 ^test. Hardy-Weinberg equilibrium was also assessed using a χ^2 ^test. A generalised linear model was used to test the differences of obesity-related anthropometric and metabolic traits between genotype groups of rs9939609 adjusted for age and gender. To correct for multiple comparisons, the false discovery rate (FDR) approach was used [21]. FDR analysis (0.05 as criteria) was applied for 15 adiposity measures simultaneously (number of tests: 15 for all, boys and girls, respectively). The association of *FTO *rs9939609 with anthropometric and metabolic traits as well as the per-allele effect size on these traits were estimated using linear regression assuming an additive genetic model. The genotypes were coded as 0, 1, or 2 corresponding to the number of copies of the risk allele (A). Logistic regression was performed to estimate the OR and 95% CI, which were calculated by Woolf's method. ORs for rs9939609 were computed assuming an additive model using multiple logistic regression adjusted for age and gender. The trend test was used for assessing the association of the SNP with obesity. The population attributable risk fraction was estimated with data from the non-obese group and was calculated as follows: *PARP*= 1-{1/[(1-*f *)^2^+2*f *(1-*f *)r+*f *^2^r^2^]}, where r is the estimated OR and *f *is the risk allele frequency [22]. The interaction of physical activity with *FTO *rs9939609 allele was detected by linear regression analysis. A general linear model was used to assess effects of physical activity and the *FTO *rs9939609 allele on BMI levels with an analysis of covariance (ANCOVA) test assuming an additive model adjusted for age and gender. The power calculation was performed using Quanto software http://hydra.usc.edu/gxe/. Our study had ≥ 75.96% to ≥ 99.84% power (enrolment of 1229 obese subjects) to detect ORs of 1.20 to 1.39 for obesity under an additive model, assuming a significance of 0.05, an allele frequency of 0.149, and obesity prevalence of 10% in the school-age children of Beijing. Thus, our case-control analysis had sufficient power to detect similar effect sizes as described by Frayling *et al. *[11]. Statistical analyses were performed with SPSS, version 13.0 (SPSS, Inc., Chicago, Illinois), and *p *< 0.05 was considered to be statistically significant.

## Results

The clinical and biometrical characteristics of the study populations are summarised in Table 1. The frequency of the rs9939609 A allele was 0.121 in the present study, and the genotypes of this SNP were in Hardy-Weinberg equilibrium in both obese and non-obese groups (*p *> 0.1). Analyses were carried out to test the association of rs9939609 with BMI and related anthropometric traits. FDR analysis (0.05 significance level) was performed to account for multiple testing. The mean values of BMI were 21.7, 22.5 and 23.0 for TT, TA and AA genotypes, respectively with a *p *value for trend of 1.39 × 10^-6^, and a BMI increase of 0.79 (95%CI 0.47 to 1.10) kg/m^2 ^per A allele, equivalent to 0.25 (95%CI 0.14 to 0.35) BMI-SDS units (Table 2). The SNP is also associated with FMP, WC, WHtR, SBP and DBP after adjusting for age and gender(*p *< 0.05 for all traits). However, the association of the SNP with SBP and DBP became weaker (both *p *> 0.05) after BMI was further adjusted. Moreover, we did not observe a significant association of the SNP with DBP, TC, TG, HDL-C, LDL-C and FPG after adjusting for age and gender (all *p *> 0.05) (Table 2).

**Table 1:** Baseline characteristics of the BCAMS study population (mean ± SD)

	All	Obese	Non-obese	*p-*value^a^
*n*	3503	1229	2274	
Boys (%)	1781 (50.8)	786 (64.0)	995 (43.8)	< 0.001
Age (years)	12.4 ± 3.1	11.8 ± 2.9	12.7 ± 3.1	< 0.001
Height (cm)	152.3 ± 15.3	152.8 ± 14.6	152.1 ± 15.7	0.18
Weight (kg)	52.4 ± 18.6	63.6 ± 18.9	46.3 ± 15.4	< 0.001
Body mass index (kg/m^2^)	21.9 ± 4.9	26.5 ± 3.7	19.4 ± 3.5	< 0.001
Body mass index-standard deviation score	1.21 ± 1.50	2.79 ± 1.01	0.35 ± 0.91	< 0.001
Waist circumference (cm)	72.4 ± 13.1	83.7 ± 10.9	66.3 ± 9.7	< 0.001
Waist-to-Height Ratio	0.47 ± 0.07	0.55 ± 0.05	0.44 ± 0.04	< 0.001
Fat mass percentage (%)	24.4 ± 8.5	30.9 ± 6.7	20.9 ± 7.3	< 0.001
Birth weight (g)	3355 ± 525	3423 ± 516	3317 ± 526	< 0.001
Systolic blood pressure (mmHg)	107.6 ± 13.9	114.3 ± 12.7	104.0 ± 13.2	< 0.001
Diastolic blood pressure (mmHg)	67.8 ± 10.0	71.9 ± 9.2	65.5 ± 9.8	< 0.001
Total cholesterol (mmol l^-1^)	4.09 ± 0.82	4.09 ± 0.74	4.10 ± 0.87	0.76
Triglycerides (mmol l^-1^)	1.03 ± 0.56	1.20 ± 0.63	0.94 ± 0.50	< 0.001
HDL-cholesterol (mmol l^-1^)	1.40 ± 0.32	1.27 ± 0.26	1.47 ± 0.33	< 0.001
LDL-cholesterol (mmol l^-1^)	2.55 ± 0.75	2.63 ± 0.66	2.51 ± 0.80	< 0.001
Fasting plasma glucose (mmol l^-1^)	5.09 ± 0.62	5.16 ± 0.48	5.06 ± 0.68	< 0.001
Regular physical activity habit^b^	56.3	53.1	58.0	< 0.001

^a ^Quantitative variables between obese and non-obese groups were assessed with a Student's *t*-test; categorical variables between groups were tested by a χ^2 ^test.

^b ^Regular physical activity was defined as participation in sports for at least 30 minutes per day and ≥ 3 days per week.

**Table 2:** Associations of the *FTO *rs9939609 variant with anthropometric and metabolic parameters (mean ± SE).

	*FTO *rs9939609 genotype^a^				
	TT	TA	AA	*p-*value for trend^a^	Estimated change unit per A allele (95% CI)^a^
All(*n*)	2718	724	61		
Height (cm)	152.3 ± 0.1	152.3 ± 0.3	153.6 ± 1.0	0.39	0.24 ((-0.33)-0.81)
Weight (kg)	51.9 ± 0.2	54.0 ± 0.5	55.6 ± 1.7	3.29 × 10^-5 b^	2.01 (1.06-2.96)
Body mass index (kg/m^2^)	21.7 ± 0.1	22.5 ± 0.2	23.0 ± 0.6	1.39 × 10^-6 b^	0.79 (0.47-1.10)
Body mass index-standard deviation score	1.15 ± 0.03	1.40 ± 0.05	1.61 ± 0.19	3.76 × 10^-6 b^	0.25 (0.14-0.35)
Waist circumference (cm)	72.0 ± 0.2	73.7 ± 0.4	75.0 ± 1.4	2.26 × 10^-5 b^	1.73 (0.93-2.54)
Waist-to-Height Ratio	0.472 ± 0.001	0.484 ± 0.002	0.488 ± 0.009	1.94 × 10^-5 b^	0.01 (0.006-0.015)
Fat mass percentage (%)	24.1 ± 0.1	25.4 ± 0.3	25.6 ± 1.0	9.75 × 10^-5 b^	1.16 (0.57-1.74)
Birth weight (g)	3358 ± 11	3345 ± 20	3342 ± 70	0.54	-12 ((-51)-27)
Systolic blood pressure (mmHg)	107.1 ± 0.2	109.2 ± 0.5	108.7 ± 1.6	1.41 × 10^-4 b ^[0.14^c^]	1.7 (0.8-2.6) [0.6((-0.2)-1.3)^c^]
Diastolic blood pressure (mmHg)	67.6 ± 0.2	68.4 ± 0.3	69.3 ± 1.2	0.01^b ^[0.63^c^]	0.8 (0.2-1.5)[0.1 ((-0.5)-0.8)^c^]
Total cholesterol (mmol l^-1^)	4.09 ± 0.02	4.10 ± 0.03	3.99 ± 0.10	0.69	-0.01 ((-0.07)-0.05)
Triglycerides (mmol l^-1^)	1.03 ± 0.01	1.04 ± 0.02	1.03 ± 0.07	0.67	0.01 ((-0.03)-0.05)
HDL-cholesterol (mmol l^-1^)	1.41 ± 0.01	1.38 ± 0.01	1.42 ± 0.04	0.11	-0.02 ((-0.04)-0.01)
LDL-cholesterol (mmol l^-1^)	2.54 ± 0.01	2.58 ± 0.03	2.41 ± 0.10	0.83	0.01 ((-0.05)-0.06)
Fasting plasma glucose (mmol l^-1^)	5.09 ± 0.01	5.10 ± 0.02	5.04 ± 0.08	0.98	0.001 ((-0.04)-0.04)

^a ^Adjusted for age and gender.

^b ^Indicates that the *p*-value remains significant after a false discovery rate control.

^c ^Adjusted for age, gender and body mass index.

Logistic regression analyses were carried out to test the null hypothesis of no association of the allelic state of rs9939609 with the risk of obesity (Table 3). The rs9939609 variant was significantly associated with obesity (*p*-value for trend = 1.12 × 10^-3^). Under an additive model, the rs9939609-A allele was associated with an increased risk of 1.29 (95%CI 1.11 to 1.50) after adjusting for age and gender. The population attributable risk fraction was lower in Chinese children and adolescents (7.07%) than previously reported in European adult populations (20.4%) [11].

**Table 3:** Associations of *FTO *rs9939609 with obesity [OR (95% CI)].

			Model 1^a^		Model 2^b^	
			
	Obese(*n *= 1229)	Non-obese(*n *= 2274)	OR (95% CI)	*p-*value for trend	OR (95% CI)	*p-*value for trend
*FTO *genotype						
TT	915	1803	1		1	
TA	288	436	1.30 (1.10-1.54)		1.31 (1.11-1.56)	
AA	26	35	1.46 (0.88-2.45)	1.09 × 10^-3^	1.46 (0.86-2.48)	1.12 × 10^-3^
Additive model						
T	2118	4042	1		1	
A	340	506	1.28 (1.11-1.49)	9.26 × 10^-4^	1.29 (1.11-1.50)	9.47 × 10^-4^

^a ^Model 1 is unadjusted.

^b ^Model 2 is adjusted for age and gender.

We have also tested the association of rs9939609 with BMI and the risk of obesity between boys and girls (see Additional file 1). Association of rs9939609 with BMI and related phenotypes such as weight, WC, WHtR and FMP are as expected in males; however, there does not appear to be a dose-dependent increase in BMI and related traits in females of the AA genotype. Further logistic analyses stratified for gender shows similar trends for the association of rs9939609 with obesity risk (see Additional file 2).

We detected an interaction between the *FTO *rs9939609 allele and physical activity that affects BMI levels (*p*_interaction _< 0.001). The effect of rs9939609 on BMI for low, moderate and severe intensity of physical activity was (0.95 ± 0.10), (0.77 ± 0.08) and (0.67 ± 0.05) kg/m^2^, respectively, when comparing A-allele with T-allele carriers.

## Discussion

Previous studies of the *FTO *gene identified a highly significant association of the common SNP rs9939609 with BMI in several European populations [11]. The association was also significant in a seven-year follow-up study in children ages 7 to 14 [11]. Subsequently, there were several replication studies in children of different ancestries [23-25]. To our knowledge, this is the first replication study of rs9939609 associations in children of Asian ancestry. Our results revealed significant associations with BMI, WC, WHtR, FMP, and weight, but not with height and birth weight. These associations indicate that the genetic variant plays a vital role in body deposition in the developmental stages beyond birth. In addition, we didn't observe statistically significant associations of the SNP with BP, glucose status or lipid traits. Furthermore, logistic regression analysis indicated that the rs9939609 variant is an independent predictor of obesity among children and adolescents. In our study, we detected a 0.79 (95%CI 0.47 to 1.10) kg/m^2 ^BMI increase per A allele, which was slightly higher than that in European children (0.4 kg/m^2 ^increase per A allele). Thus, the effect size of the rs9939609 A allele on the risk of obesity is comparable to that in populations of European children [11].

In Asian adult populations, replication studies have yielded inconsistent results [12-15]. This discrepancy might partly be due to the low minor allele frequency (MAF) of the variant allele, and therefore there is not enough power to dissect the genetic contribution to obesity. The MAF of rs9939609 was substantially lower in the present study (12.1%) than in European populations (~45%) [11]. A meta-analysis combining all studies in East Asian populations showed that the *FTO *rs9939609 polymorphism is associated with obesity [26]. Other reasons might be differences in sample collection, varying BMI standards, population admixture or environmental exposure. For example, Andreason *et al. *[27] reported that physical activity might attenuate the effects of the *FTO *rs9939609 variant on BMI. We also found an interaction between the *FTO *variant and self-reported physical activity in our study. However, the study in Finnish children (*n *= 438) did not show an interaction between the *FTO *variant and leisure-time physical activity [28]. These conflicting results might be due to low statistical power or inaccurate measurements of physical activity. Therefore, population-based studies with large sample sizes and a more direct measure of physical activity are necessary to further investigate this association.

Recently, Jacobsson *et al. *[29] reported a major gender differences in the association of *FTO *gene with obesity-related phenotypes in severely obese European children. In our study, there was also a gender difference in the association of this SNP with obesity, though the trends were not consistent between our population and the population of European children. Possible reasons for gender differences may be the low MAF in children of Chinese ancestry and small sample sizes in subgroups stratified for gender. Other reasons could be fat distribution, deposition and accumulation in overall body composition are different between boys and girls [30]. Further large scale studies are needed to explore possible gender effects.

The mechanisms of how *FTO *variants influence BMI and obesity-related traits are unclear. The FTO protein was highly expressed in the brain (particularly the hypothalamus), which is consistent with an important role of the central nervous system in regulating weight [31]. A recent report by a British group [23] indicated that the rs9939609 variant influences energy-dense food intake rather than regulation of energy expenditure in children. *Andreasen et al*. [27] reported that fasting serum leptin levels were significantly elevated in rs9939609 A allele carriers. Other studies have supported a central role of *FTO *on cerebrocortical insulin sensitivity because individuals homozygous for the risk allele have a reduced insulin response in the brain [32]. Future studies focusing on these phenotypes might help clarify the mechanisms through which common variants of the *FTO *gene and obesity are related.

The strengths of this study included the population-based case-control study design and a high response rate that minimised selection bias. In addition, our study had sufficient power to detect the association of rs9939609 with obesity in Chinese children and adolescents. However, several limitations should also be noted. First, the case-control study design does not allow for a causality conclusion to be made. Second, the low MAF of rs9939609 in this Chinese population impedes subgroup analysis (e.g., gender) with sufficient power. Third, only one SNP of *FTO *was measured in this study, so it is unclear whether the identified association is due to this specific sequence variant or to another variant in tight linkage disequilibrium with rs9939609 [28].

## Conclusions

In conclusion, our findings detected an association between the *FTO *rs9939609 variant and BMI and risk of obesity among children and adolescents of Chinese ancestry. The role of rs9939609 in mediating susceptibility to a higher BMI or an increased risk of obesity among children requires further investigations including detailed environmental exposure studies, especially with respect to energy intake and food choices. In addition, further understanding of the linkage disequilibrium pattern of the *FTO *gene in different populations may aid in the search of casual gene for obesity.

## Competing interests

The authors declare that they have no competing interests.

## Authors' contributions

JM and XYW designed the study, collected and interpreted the data, drafted the manuscript and made critical revisions. BX and YS participated in genotyping, analysed and interpreted the data, drafted the manuscript and made critical revisions. MXZ, XL, XYZ and LJW participated in genotyping. HC and DQH participated in collecting the data. KL and LSL made critical revisions. All the authors read and approved the final manuscript.

## Pre-publication history

The pre-publication history for this paper can be accessed here:

http://www.biomedcentral.com/1471-2350/11/107/prepub

## Supplementary Material

Additional file 1 **Associations of *FTO *rs9939609 with anthropometric parameters (mean ± SE). stratified for gender**. Click here for file

Additional file 2 **Associations of *FTO *rs9939609 with obesity stratified for gender [OR (95% CI)]**. Click here for file
