# Supplementary material for: The common rs9939609 variant of the fat mass and obesity-associated gene is associated with obesity risk in children and adolescents of Beijing, China
Source: BMC Med Genet. 2010 Jul 5;11:107. doi: 10.1186/1471-2350-11-107 (PMC2914647; doi:10.1186/1471-2350-11-107)
Supplement: Additional file 1 — Associations of FTO rs9939609 with anthropometric parameters (mean ± SE). stratified for gender. [file 1471-2350-11-107-S1.DOC]

**Additional file 1**

**Associations of FTO rs9939609 with anthropometric parameters (mean ± SE) stratified for gender.**

|  | FTO rs9939609 genotype ^a^ | | | *p-*value for trend ^a^ | Estimated change unit per A allele (95% CI) ^a^ |
| --- | --- | --- | --- | --- | --- |
|  | TT | TA | AA |  |  |
| Boys（*n*） | 1380 | 368 | 33 |  |  |
| Height (cm) | 154.2 ± 0.2 | 154.1 ± 0.4 | 154.7 ± 1.3 | 0.99 | 0.002 ((-0.77)-0.77) |
| Weight (kg) | 55.8 ± 0.4 | 57.0 ± 0.7 | 61.2 ± 2.5 | 0.02 ^b^ | 1.70 (0.28-3.13) |
| Body mass index (kg/m^2^) | 22.6 ± 0.1 | 23.1 ± 0.2 | 24.9 ± 0.8 | 3.01×10^-3 b^ | 0.71 (0.24-1.18) |
| Body mass index -standard deviation score | 1.40 ± 0.04 | 1.58 ± 0.08 | 2.20 ± 0.27 | 1.54 ×10^-3 b^ | 0.25 (0.09-0.40) |
| Waist circumference (cm) | 75.7 ± 0.3 | 76.8 ± 0.6 | 80.5 ± 2.2 | 0.01 ^b^ | 1.52 (0.29-2.75) |
| Waist-to-Height Ratio | 0.49 ± 0.002 | 0.50 ± 0.004 | 0.52 ± 0.013 | 7.72×10^-3^ ^b^ | 0.010 (0.003-0.017) |
| Fat mass percentage (%) | 23.1 ± 0.2 | 23.6 ± 0.4 | 25.6 ± 1.4 | 0.07 | 0.72 (-0.05-1.50) |
| Birth weight (g) | 3419 ± 15 | 3377 ± 29 | 3404 ± 96 | 0.26 | -31 ((-87) -24) |
| Systolic blood pressure (mmHg) | 110.0 ± 0.3 | 111.9 ± 0.7 | 114.4 ± 2.3 | 2.83×10^-3^ ^b^  [0.12 ^c^] | 2.0 (0.7-3.2)  [0.8 ((-0.2)-1.9) ^c^] |
| Diastolic blood pressure (mmHg) | 68.5 ± 0.3 | 69.4 ± 0.5 | 71.0 ± 1.7 | 0.05  [0.46 ^c^] | 1.0 (0.01-1.9)  [0.3((-0.5)-1.2) ^c^] |
| Total cholesterol (mmol l^-1^) | 4.08 ± 0.02 | 4.03 ± 0.04 | 3.95 ± 0.13 | 0.17 | -0.05 ((-0.13)-0.02) |
| Triglycerides (mmol l^-1^) | 1.02 ± 0.01 | 1.01 ± 0.03 | 1.11 ± 0.10 | 0.85 | 0.01 ((-0.05)-0.06) |
| HDL-cholesterol (mmol l^-1^) | 1.39 ± 0.01 | 1.37 ± 0.02 | 1.37 ± 0.05 | 0.25 | -0.02 ((-0.05)-0.01) |
| LDL-cholesterol (mmol l^-1^) | 2.54 ± 0.02 | 2.52 ± 0.04 | 2.39 ± 0.12 | 0.36 | -0.03 ((-0.10)-0.04) |
| Fasting plasma glucose (mmol l^-1^) | 5.15 ± 0.01 | 5.18 ± 0.03 | 5.09 ± 0.10 | 0.58 | 0.01((-0.04)-0.07) |
|  |  |  |  |  |  |
| Girls（*n*） | 1338 | 356 | 28 |  |  |
| Height (cm) | 150.3 ± 0.2 | 150.6 ± 0.4 | 152.9 ± 1.4 | 0.13 | 0.60 ((-0.17)-1.37) |
| Weight (kg) | 47.9 ± 0.3 | 51.0 ± 0.6 | 49.8 ± 2.2 | 3.84×10^-5 b^ | 2.47 (1.30-3.65) |
| Body mass index (kg/m^2^) | 20.8 ± 4.2 | 21.9 ± 4.2 | 20.9 ± 4.6 | 6.12×10^-5 b^ | 0.88 (0.45-1.30) |
| Body mass index -standard deviation score | 0.89 ± 0.04 | 1.22 ± 0.07 | 0.99 ± 0.26 | 6.73×10^-4 b^ | 0.25 (0.10-0.39) |
| Waist circumference (cm) | 68.1 ± 0.3 | 70.6 ± 0.5 | 69.2 ± 1.9 | 8.64×10^-5 b^ | 2.03 (1.02-3.04) |
| Waist-to-Height Ratio | 0.45 ± 0.002 | 0.47 ± 0.003 | 0.45 ± 0.011 | 5.26×10^-4 b^ | 0.011(0.005-0.017) |
| Fat mass percentage (%) | 25.1 ± 0.2 | 27.1 ± 0.4 | 25.5 ± 1.6 | 3.37×10^-4 b^ | 1.55(0.71-2.40) |
| Birth weight (g) | 3298 ± 15 | 3313 ± 28 | 3279 ± 103 | 0.77 | 8((-47)-63) |
| Systolic blood pressure (mmHg) | 104.1 ± 0.3 | 106.6 ± 0.6 | 102.6 ± 2.2 | 0.01 ^b^  [0.45 ^c^] | 1.6 (0.4-2.8)  [0.4((-0.6)-1.5) ^c^] |
| Diastolic blood pressure (mmHg) | 66.5 ± 0.2 | 67.3 ± 0.5 | 67.5 ± 1.7 | 0.12  [0.99 ^c^] | 0.7 ((-0.2)-1.7)  [-0.1((-0.9)-0.8) ^c^] |
| Total cholesterol (mmol l^-1^) | 4.11 ± 0.02 | 4.17 ± 0.05 | 4.02 ± 0.16 | 0.51 | 0.03 ((-0.06)-0.12) |
| Triglycerides (mmol l^-1^) | 1.03 ± 0.01 | 1.06 ± 0.03 | 0.93 ± 0.10 | 0.66 | 0.01 ((-0.04)-0.07) |
| HDL-cholesterol (mmol l^-1^) | 1.42 ± 0.01 | 1.39 ± 0.02 | 1.46 ± 0.06 | 0.21 | -0.02 ((-0.05)-0.01) |
| LDL-cholesterol (mmol l^-1^) | 2.55 ± 0.02 | 2.63 ± 0.04 | 2.44 ± 0.15 | 0.27 | 0.05 ((-0.04)-0.13) |
| Fasting plasma glucose (mmol l^-1^) | 5.04 ± 0.02 | 5.02 ± 0.04 | 5.00 ± 0.13 | 0.66 | -0.02 ((-0.08)-0.05) |

^a^ Adjusted for age.

^b^ Indicates that the *p*-value remains significant after a false discovery rate control.

^c^ Adjusted for age and body mass index.
