# Supplementary material for: The common rs9939609 variant of the fat mass and obesity-associated gene is associated with obesity risk in children and adolescents of Beijing, China
Source: BMC Med Genet. 2010 Jul 5;11:107. doi: 10.1186/1471-2350-11-107 (PMC2914647; doi:10.1186/1471-2350-11-107)
Supplement: Additional file 2 — Associations of FTO rs9939609 with obesity stratified for gender [OR (95% CI)]. [file 1471-2350-11-107-S2.DOC]

**Additional file 2**

**Associations of *FTO* rs9939609 with obesity stratified for gender [OR (95% CI)].**

|  | Obese | Non-obese | Model 1 ^a^ | | Model 2 ^b^ | |
| --- | --- | --- | --- | --- | --- | --- |
|  |  |  | OR (95% CI) | *p-*value for trend | OR (95% CI) | *p-*value for trend |
| Boys (*n*=1781) |  |  |  |  |  |  |
| *FTO* genotype |  |  |  |  |  |  |
| TT | 592 | 788 | 1 |  | 1 |  |
| TA | 173 | 195 | 1.18 (0.94-1.49) |  | 1.17 (0.93-1.48) |  |
| AA | 21 | 12 | 2.33 (1.14-4.77) | 0.018 | 2.34 (1.14-4.80) | 0.020 |
| Additive model |  |  |  |  |  |  |
| T | 1357 | 1771 | 1 |  | 1 |  |
| A | 215 | 219 | 1.28 (1.05-1.57) | 0.016 | 1.27 (1.04-1.56) | 0.018 |
|  |  |  |  |  |  |  |
| Girls (*n*=1722) |  |  |  |  |  |  |
| *FTO* genotype |  |  |  |  |  |  |
| TT | 323 | 1015 | 1 |  | 1 |  |
| TA | 115 | 241 | 1.50 (1.16-1.93) |  | 1.53 (1.18-1.99) |  |
| AA | 5 | 23 | 0.68 (0.26-1.81) | 0.024 | 0.70 (0.26-1.88) | 0.018 |
| Additive model |  |  |  |  |  |  |
| T | 761 | 2271 | 1 |  | 1 |  |
| A | 125 | 287 | 1.30 (1.04-1.63) | 0.023 | 1.32 (1.05-1.67) | 0.017 |

^a^  Model 1 is unadjusted.

^b^  Model 2 is adjusted for age.
